# Advanced Image Analytics for Mobile Robot-Based Condition Monitoring in Hazardous Environments: A Comprehensive Thermal Defect Processing Framework

**DOI:** 10.3390/s24113421

**Published:** 2024-05-26

**Authors:** Mohammad Siami, Tomasz Barszcz, Radoslaw Zimroz

**Affiliations:** 1 AMC Vibro Sp. z o.o., Pilotow 2e, 31-462 Kraków, Poland; 2 Faculty of Mechanical Engineering and Robotics, AGH University, Al. Mickiewicza 30, 30-059 Kraków, Poland; tbarszcz@agh.edu.pl; 3 Faculty of Geoengineering, Mining and Geology, Wrocław University of Science and Technology, Na Grobli 15, 50-421 Wrocław, Poland; radoslaw.zimroz@pwr.edu.pl

**Keywords:** CNN, VGG16, random forest, XGBoost, semantic segmentation, PCA-K-means, thermal imaging, belt conveyor, thermal defects

## Abstract

In hazardous environments like mining sites, mobile inspection robots play a crucial role in condition monitoring (CM) tasks, particularly by collecting various kinds of data, such as images. However, the sheer volume of collected image samples and existing noise pose challenges in processing and visualizing thermal anomalies. Recognizing these challenges, our study addresses the limitations of industrial big data analytics for mobile robot-generated image data. We present a novel, fully integrated approach involving a dimension reduction procedure. This includes a semantic segmentation technique utilizing the pre-trained VGG16 CNN architecture for feature selection, followed by random forest (RF) and extreme gradient boosting (XGBoost) classifiers for the prediction of the pixel class labels. We also explore unsupervised learning using the PCA-K-means method for dimension reduction and classification of unlabeled thermal defects based on anomaly severity. Our comprehensive methodology aims to efficiently handle image-based CM tasks in hazardous environments. To validate its practicality, we applied our approach in a real-world scenario, and the results confirm its robust performance in processing and visualizing thermal data collected by mobile inspection robots. This affirms the effectiveness of our methodology in enhancing the overall performance of CM processes.

## 1. Introduction

Condition-based maintenance (CBM) facilitated by mobile robots constitutes a pivotal technological advancement with broad implications across industries confronting challenging environmental conditions, such as mining. Modern inspection robots present a viable solution for executing diverse inspection tasks within austere and inaccessible environments for human operators. Employing inspection robots for the monitoring of industrial infrastructure holds the potential to mitigate incidents during routine inspections. Additionally, the automation of inspection procedures using robots enhances the longevity of machinery, as these devices excel in precision and efficiency in performing CM tasks, as evidenced by previous research [1,2].

These inspection robots are equipped with a myriad of sensors, encompassing RGB and infrared (IR) imaging, auditory capabilities, Light Detection and Ranging (LiDAR) technology, and gas hazard sensors [3,4,5,6,7].

In recent years, advancements in camera technology and data processing have led to an increased emphasis on CM utilizing camera data [8,9]. This non-contact approach has proven advantageous in specific applications, with notable progress observed in the past decade, underscoring its high potential for widespread industrial application. This study specifically focuses on the application of IR imaging for CM of belt conveyor (BC) idlers, acquired through the deployment of mobile robots in authentic mining environments. BCs serve as the primary mode of horizontal bulk material transportation at mining sites. Regular inspection of various components is imperative to minimize the risk of sudden breakdowns within BC networks. Compliance with internal regulations at mining sites necessitates daily inspections of different BC components. The extensive length of BCs, spanning thousands of meters, coupled with the need to individually inspect dozens of components under harsh environmental conditions, underscores the complexity of this task [10,11,12,13,14,15,16].

Monitoring the thermal conditions of idlers assumes paramount importance, as overheated idlers pose a fire hazard and can cause severe damage to BC networks. Given the multitude of IR images captured by mobile robots during inspection tasks, the associated challenge lies in developing an effective industrial big data analytics methodology for processing this wealth of information.

In recent years, the challenges associated with image-based CM methods in the context of industrial big data have prompted the exploration of various deep learning approaches. Notably, Convolutional Neural Network (CNN) architectures based on U-Net have gained prominence, owing to their efficacy in semantic segmentation tasks within image processing applications [17,18]. Despite the success of U-Net, its performance may be significantly constrained in scenarios with a limited number of training samples.

To address this limitation, we propose a hybrid segmentation method designed to overcome the challenges encountered by U-Net-based architectures in datasets characterized by significant imbalance and scarcity of samples. In this approach, we leverage the pre-trained VGG16 architecture developed by the visual geometry group from Oxford to extract pertinent features from pre-processed IR image samples. Subsequently, to classify the extracted features and delineate anomalies within the studied samples, RF and XGBoost methods are employed. To train the classifiers, the features extracted by the CNN are used as the input data for the classifiers. The classification models are trained using a small subset of positive pre-processed samples, and the results are compared with different U-Net architectures.

The classification of unlabeled, segmented IR image samples serves to visualize essential features that aid in clustering overheated idlers based on the degree of severity. In this paper, we propose a methodology based on Principal Component Analysis (PCA) and K-means for clustering of unlabeled, segmented thermal anomalies in captured IR images. Our results indicate that the suggested methodology facilitates accurate classification of thermal anomalies into distinct classes.

The contributions of the proposed framework are summarized as follows:

- A hybrid model based on the pre-trained VGG16 CNN architecture as a feature extractor and RF and XGBoost as classifiers was introduced to industrial CM to perform semantic segmentation tasks on IR image datasets.
- The employed unsupervised approach using PCA-K-means could help us significantly reduce the dimensions of the unlabeled segmented labels to cluster them based on degree of severity.
- A benchmark of the proposed models in segmentation and classification of thermal defects in BC idlers was created on an industrial dataset, proving the usability of the created data, as well as the model in the other industrial IR image-based CM domains.

The subsequent sections of the paper are structured as follows: The literature review presented in Section 2 comprehensively discusses various types of damage leading to bearing failure in BC idlers. Additionally, it covers relevant studies on IR image processing methods for CM purposes, encompassing both classical and deep learning-based approaches. Section 3 provides a detailed exposition of our proposed methodology. In Section 4, the selected metrics for evaluating the proposed image processing pipeline are discussed. Section 5 presents the experimental results and their analysis. Finally, Section 6 draws conclusions based on the findings presented in this study.

## 2. Literature Review

The application of mobile inspection robots for CM of industrial infrastructure located at mining sites dates back to the 1990s [19,20]. Automated anomaly detection and classification methods can be categorized into non-learning approaches, which usually use classical image processing methods to extract patterns in captured IR images, and machine and deep learning approaches, which are data analysis-based methods for the automation of analytical model building. Before discovering the main advantage of the mentioned methods, we first point out the main reasons that can cause bearing failures in BC idlers in the next section.

### 2.1. Failure Causes in Idler Bearings

Idlers are an important part of BCs that are responsible for the movement of the rubber belt and the transport of materials [21,22,23]. Each idler consists of two bearings. Different types of damage can cause bearing failure. There are six types of faults these are plastic deformation, corrosive wear, fretting corrosion, fractures, surface fatigue wear (pitting), and abrasive and adhesive wear [24].

Plastic deformation might be considered the most important factor in bearing failure, which can occur due to uneven loads or improper assembly. In such a case, grease is squeezed from the bearing contact surfaces, which causes direct metal-to-metal contact that leads to overheating and sudden damage to the idler [25,26].

Corrosive wear is a type of fault that occurs mainly in bearings with poor sealing. Bearings in BC idlers can be constantly exposed to extreme weather conditions. Most of the less expensive idlers are not protected from abrasive particles. These substances, such as dirt, snow, rain, dust, etc., can reach inside the idlers and be mixed up with lubricants, which increases the friction forces between the interior surfaces of the bearing [27,28].

Fretting corrosion arises in instances where there is a separation between the subsequent components: the shaft and the inner ring, the housing and the outer ring, the rolling bodies and the cage, or the rolling bodies and the rings. The gap induces relative motion between the parts, resulting in the detachment of minuscule particles when there are strong vibrations and no lubricant layer present. For this reason, the cavities created by fretting corrosion become filled with fine-particulate wear debris. As the wear process progresses, the pits combine.

Fracture-related damage can be caused by forced (overload) or fatigued (material fatigue) fractures. Forced fractures might occur due to local overload, high-energy impacts, and excessive pressures. Fatigue fractures might occur during daily operations. In cage fracture, the spacing between the rolling elements is reduced, which causes the bearing balls to start touching each other, which makes the idlers start rotating eccentrically. The broken bearing will eventually stop the idler’s movement. The friction forces can heat up the idlers up to 450 °C, which might cause a fire in the production line [14].

Surface fatigue wear or pitting can be caused by constant cyclic stress on bearing contact surfaces during daily operations. It starts with small cracks on the bearing surface; afterward, the crack will expand, eventually creating pits of different sizes that cause serious damage to the internal surfaces of the bearing [25].

Abrasive and adhesive wear can be caused by damage in the form of grooves, cuts, and scratches in the bearing contact surfaces. If hard particles enter the contact zone during operating time, such damage can occur. Adhesive wear can only happen on bearings that are poorly lubricated. In such cases, the adhesive forces can generate heat, where microwelding occurs instantaneously [26].

### 2.2. Classical IR Image Processing Methods

Due to the advantages of non-contact measurement methods, IR imaging has become an important part of preventive maintenance programs. IR imaging enables us to visualize and verify thermal anomalies (hotspots) and determine the severity of defects. Furthermore, it can help inspectors establish a time frame within which a machine should be repaired. In this direction, different researchers have worked on this idea in recent years.

There are two main approaches for analyzing IR images, which are referred to as quantitative and qualitative methods. In quantitative approaches, the exact temperature of the studied object should be taken into account. As measurement of the exact temperature of objects can be difficult or inaccurate in some applications due to environmental factors and the object emissivity value, the application of quantitative methods for CM purposes that take place in real-life scenarios can be limited. However, in the qualitative method, characteristic patterns of the relative temperatures of objects are studied in comparison to their environment are measured. Qualitative analysis methods are popular for CM of industrial infrastructure. The severity or level of overheating can be defined on the basis of the difference in an object’s temperature in comparison to its environment [29,30,31,32].

The process of identifying damage for CM purposes is known as structural health monitoring, where the imposed damage (in our case, thermal faults) is defined as changes to the normal behavior of the materials’ temperature [33]. IR imaging methods can help supervisors detect thermal defects in different fields, including the aerospace, civil, and mechanical engineering infrastructure industries.

For automatic detection of thermal defects and assessment of idler conditions in BC networks, Szrek et al. employed classical image processing methods, including canny edge detection and blob detection techniques, for the identification of thermal anomalies within captured IR images. However, due to the nature of the classical method, the performance of the proposed method can be affected by the proportion of false-positive samples in the final results [34].

To improve detection results in IR images with complex backgrounds, Siami et al. proposed an automatic segmentation method for excluding overheated idlers (hot surfaces) from the background (cold surfaces). However, the presence thermal sources unrelated to idlers in mining sites within the captured IR image in some frames caused over-segmentation [35].

Similarly, in [36], the authors proposed a histogram analysis method for the identification of hotspots in IR images captured from photovoltaic modules. For this reason, the authors extracted different statistical features from captured images to identify areas in the images representing thermal defects. However, they only analyzed a limited number of IR images, which makes their methodology unsuitable for the identification of thermal defects in large datasets consisting of many samples. In conclusion, one can notice that, due to the nature of traditional image processing methods, they are not a proper answer for automatic fault detection for CM purposes on large scales.

### 2.3. Deep Learning-Based IR Image Processing Methods

The application of robotics-based CM methods has improved day by day as a result of rapid developments in machine learning methods. Since 2012, the application of deep learning methods for image processing tasks, including object localization, detection, segmentation, and classification in images with complex backgrounds, has received attention [37]. Inspired by CNNs, different architectures have been developed in the past decade. Previously, researchers relied on handcrafted features to perform automated machine vision tasks. For instance, support vector machines, edge features, and adaptive boosting are worked in this way [18,38,39].

The feature extraction methods that have been developed based on CNN architectures can be categorized into region-based classification and semantic segmentation methods [40]. The region-based classification methods rely on the definition and classification of regions that include desirable objects. The major drawback of region-based classification methods is that extracted features are only recognized within a captured scene, so we cannot accurately define the size and greatness of the identified features. However, in semantic segmentation methods, the presented pixels in a captured image are assigned to a pre-defined class. Therefore, pixel-level classification enables us not only to find the exact location of faults but also to determine their size and severity.

In region-based methods, a certain region in the processed image is assigned to a possible pre-defined feature. In our previous work [41], we proposed a binary classification method based on a CNN architecture to classify frames with signs of thermal defects on BC idlers. However, due to the limitations of the region-based classification method, we could not cluster the defected idlers based on their degree of severity. Similarly, in [42], the authors proposed a region-based classification method for the identification of hotspots in photovoltaic panels. The employed CNN architecture was used for the classification of regions in images with signs of thermal anomalies. Rahman et al. proposed a hybrid method that uses a pre-trained CNN architecture (VGG16) as a feature extractor and XGBoost as a classifier to perform region-based image classification to detect tuberculosis from chest X-rays [43]. Their work demonstrated that using a pertained CNN as a feature extractor and replacing the roll of the fully connected layer with an XGBoost classifier can significantly reduce the number of samples necessary to train the model.

Semantic segmentation methods aim to classify each pixel into predetermined classes for the extraction of the exact locations of desired features. Despite recent progress in the application of semantic segmentation models to regular images, a limited number of researchers have studied the importance of deep learning-based IR image processing approaches for the monitoring of challenging environments.

In this direction, the authors of [44] proposed a segmentation model applied to IR images of carbon fiber-reinforced polymer (CFRP) to identify subsurface defects. The researcher suggested state-of-the-art semantic segmentation methods, including DeepLabV3+ and U-Net models, that can outperform older ML methods such as RF or support vector machine (SVM). Similarly, the work of Pozzer et al. investigated the performance of different deep learning architectures for the identification of concrete anomalies using IR and regular imaging approaches [45]. Their results showed that the MobileNetV2 model achieved promising performance, identifying 79.7% of the total defects in IR images of highly damaged concrete areas. In the same direction, the authors of [46] proposed a hybrid model using a pre-trained CNN (VGG16) model as a feature extractor and multiple sets of dilated convolutions to make the network gain a large enough receptive field. At the end, A multilayer perceptron (MLP) fuses the multi-scale convolution features and predicts each pixel to segment the desired target. However, considering the complexity of the provided model, it did not provide a huge advantage compared to other studied architectures in terms of accuracy and computational time aspects.

The main drawback of the reviewed paper can be attributed to the lack of a proper segmentation method in an unbalanced dataset with a limited number of positive samples, since, in most cases, deep learning models need to have access to a large number of samples to be successfully trained. Furthermore, in the proposed methodologies, researchers did not study a procedure to estimate the severity of the damage by analyzing the segmented frames. Moreover, researchers have not proposed a proper dimension reduction methodology for visualization of the gathered industrial big data in an efficient way to be understood by managers and supervisors for industrial applications.

## 3. Proposed Methodology

**Figure 1:**
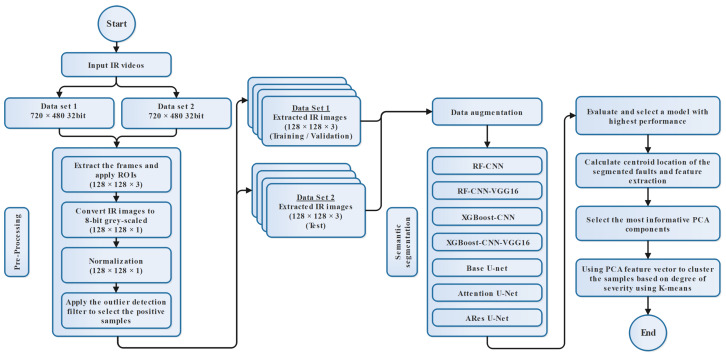
Simplified flowchart of proposed IR image processing pipeline.

In this section, the key elements of the proposed methodology are described. A general flowchart of the procedure is presented in Figure 1. As part of the pre-processing steps, the overall quality of the datasets is improved. This is done before the models are trained and tested. Moreover, we describe the related procedures for training the studied models and select the best result for clustering of the segmented thermal faults. Finally, the associated steps for dimension reduction procedures and clustering are described.

### 3.1. Pre-Processing

The complexity of the background around BC systems, which includes sun reflections, moving workers, and other overheated objects in mining sites, brings much difficulty to the accurate detection of overheated idlers. Therefore, it is critical to consider a series of pre-processing steps in order to reduce the computational cost. To analyze the captured IR videos, first, we extract the frames and store them for future pre-processing tasks. To enhance the pixel intensity and overall quality of captured frames, three different pre-processing methods are carried out on captured datasets, including region of interest (ROI) definition, color transformation, and data normalization.

The mobile robot is equipped with an IR camera that is pointed toward BC systems to capture IR videos from idlers. Sequences of frames are captured from IR videos that let us accurately localize the idlers. To reduce computation consumption and to raise the precision of detection, a square-shaped ROI with a size of 128×128 pixels is defined on the original frames [35,41].

Moreover, to reduce the computational burden during training of the studied models and improve the accuracy of segmentation results, we decided to convert colored, 16-bit IR images images (128×128×3) to grayscale, 8-bit images (128×128×1), as shown in Equation (1).

(1) $$I_{\mathrm{gray}\;}=0.299\times I_{red}+0.587\times I_{\mathrm{green}}+0.114\times I_{blue}$$

The intensity values or, in other words, the relative temperature of each pixel in comparison to its neighboring pixels, are pre-defined by the IR camera within the frame borders. To define the statistical parameters that work well for analyzing ROIs, pixel intensity values are recalculated with respect to the pixel distribution in defined ROIs by normalizing the grayscale ROIs [47].

The normalization process transforms the exemplary grayscale IR image with an n-dimensional $I:\left\{X\subseteq R^{n}\right\}\to \left\{{\mathrm{Min}}_{\mathrm{Original}},\ldots ,{\mathrm{Max}}_{\mathrm{Original}}\right\}$, with intensity values in the range of $\left(Min_{Orginal},Max_{Orginal}\right)$ into a normalized image $I:\left\{X\subseteq R^{n}\right\}\to \left\{{\mathrm{Min}}_{\mathrm{ROI}},\ldots ,{\mathrm{Max}}_{\mathrm{ROI}}\right\}$ with intensity values in the range of $\left(Min_{ROI},Max_{ROI}\right)$. By considering the linear relationship between $I_{Orginal}$ and $I_{ROI}$, the normalization with the pre-defined grayscale IR images can be performed as follows:

(2) $$I_{N}=\left(I-{\mathrm{Min}}_{\mathrm{Original}}\right)\frac{Max_{ROI}-Min_{ROI}}{{\mathrm{Max}}_{\mathrm{Original}}-{\mathrm{Min}}_{\mathrm{Original}}}+Min_{ROI}$$

### 3.2. Handling the Class Imbalance Problem Using an Outlier Detection Filter

Table 1 describes the percentage of extracted frames that contain overheated idlers (positive samples) in comparison to healthy samples. One can notice that the percentage of positive samples was, by far, less than the percentage of negative samples. These numbers indicate that our original dataset suffers from the class imbalance problem, which can considerably affect the performance of the semantic segmentation model in the correct detection of overheated idlers. In this way, training deep learning models can become a crucial issue [48].

Different methods, including initial filtering using classical image processing methods, can help us identify and eliminate unnecessary samples (negative samples) to address the class imbalance problem by creating a dataset that only consists of positive samples. In our case, we decided to remove the majority of negative samples to successfully train the studied deep learning models on positive samples. To do so, we employed our previous study to initially remove the negative samples from the input datasets [35]. This process helps us reduce the number of unwanted samples and, consequently, the computational complexity of training the deep learning models.

In the outlier detection method, we defined the thermal anomalies as hotspots within the extracted frames. To define the anomalies, the interquartile range (IQR) can be used to extract histogram features to define the optimal threshold value for separating the hotspots from the background. After the initial segmentation, we cluster the samples into two groups of positive and negative samples. Therefore, in the next level, only samples with signs of thermal anomalies are saved for the training, testing, and validation of the studied models.

### 3.3. Data Augmentation

Oversampling and undersampling are the most common techniques for addressing model overfitting and class imbalance issues in image datasets. Data augmentation can be considered an oversampling method to amplify the minority classes [49,50,51].

**Figure 2:**
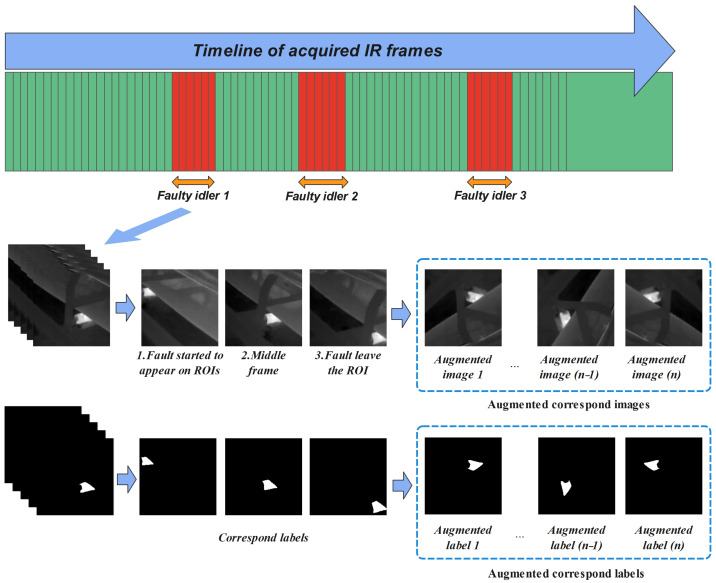
Data augmentation on IR image samples and corresponding ground-truth labels.

The image augmentation techniques can be divided into the following three different categories: geometric and color space transformations and pixel point operations. In this work, different data augmentation techniques, namely vertical flip, random 90-degree rotation, horizontal flip, and transpositions, were applied to IR image datasets (Figure 2).

### 3.4. Efficient Image Feature Extraction Using CNN and Transfer Learning Approach

Inspired by the human visual cortex, CNNs were first introduced in 1980. In a CNN architecture, the convolutional layers are considered the main blocks that set convolutional operators on the input data, which include element-wise multiplications and summations [52,53].

The first objective of this work is to study CNN architectures that can be efficiently trained with a small number of annotated image samples. This approach is especially helpful when using a highly imbalanced dataset with a small number of positive samples with complex scenes. CNN architectures are usually used in computer vision problems due to their ability to perform different image processing tasks, including pixel-level semantic segmentation.

**Figure 3:**
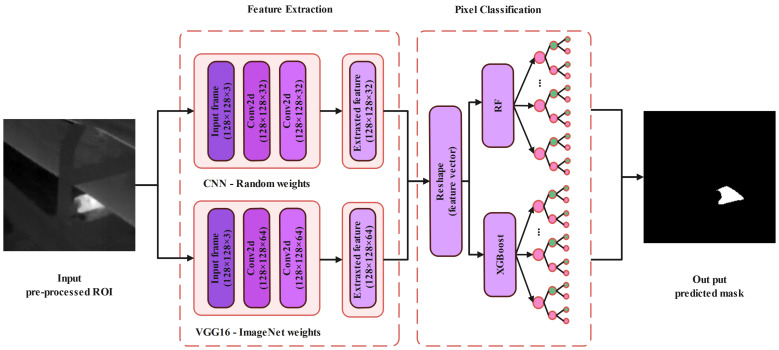
The architecture of proposed CNNs to efficiently extract the pixel features and classify them to perform semantic segmentation tasks.

The initial part of CNN architectures, called feature extraction networks, consists of neural networks that are responsible for extracting unique features from the input image. Later, the extracted features are processed through a classification model to generate the desired output (Figure 3). The basic building blocks of the initial part of the CNN architecture can be defined as follows:

- Input layer
  In the input layer, the image is entered into the CNN architecture. In examples where the initial model has been trained on samples with different dimensions, to take advantage of the transfer learning approach, the input images need to be reshaped to match the pre-trained CNN architecture input dimensions.
  In our case, to reshape the dimension of the input images, we first reduce the number of image channels from three (RGB) to one (grayscale). However, to take advantage of the pre-trained VGG16 architecture without the need to transform the 8-bit grayscale images into color ones, we create two new dimensions and repeat the image array. As long as we have the same image over all three channels, the performance of the model should be the same as when it was initially trained on RGB images.
- Convolution layer
  In the convolutional layer, the convolutional filter, also called the kernel, is defined as a matrix that passes over the input sample to generate feature maps. The generated feature maps in the convolutional layer accentuate the special features of the input image. Convolution is a linear operation that is used in different domains, such as image processing, statistics, and physics.
  A mathematical operation called a convolutional can be defined by sliding the kernel matrix over the input sample’s matrix. The element-wise matrix multiplication should be performed at every pixel to sum the result and represent it in a feature map.
  To perform feature map extraction in RGB images, usually, multiple 2D convolutional filters are employed in CNN architectures. The feature in this process is extracted by computing the 2D convolutional filter from the input sample channels.
  Considering an RGB sample (three channels), the size of the input image can be defined as $N_{1}\times N_{1}$, where $m_{1}$ 2D convolutional kernels of size $K_{1}\times K_{1}$ are available in the first 2D convolutional layer. Therefore, the first 2D convolutional layer can generate the $m_{1}$ feature maps of size $\left(N_{1}-k_{1}+1\right)$ × $\left(N_{1}-k_{1}+1\right)$. Each feature map is calculated based on computation of the dot product between the weight matrix, which is defined by ω, and the local area position (x,y) and the neuron $V_{ij}^{xy}$ value at position (x,y) in the *j*th layer, as follows:
  (3) $$V_{ij}^{xy}=\sigma \left(b_{ij}+\sum_{m}\sum_{k=0}^{K_{i}-1}\sum_{p=0}^{P_{i}-1}w_{ij\;m}^{kp}V_{(i-1)m}^{\left(x+k)(p+p\right)}\right)$$
  In Equation (3), σ(.) defines the activation function of the *i*th layer, while $b_{ij}$ is an additive bias of the *j*th feature map at the *i*th layer. Moreover, variable *m* indexes the connection between the feature map in the (i−1)th layer and the current *j*th feature map. Moreover, Ki and Pi refer to the height and width of the 2D convolutional kernel, respectively. The variable $\omega_{ijm}^{kp}$ is the weight for input $V_{(i-1)m}^{\left(x+k)(y+p\right)}$ with an offset of (k,p) in the 2D convolutional kernel [54,55].
- Activation function
  The activation function is a component that takes place after the convolutional layer. The generated feature maps in the convolutional filter layer should be processed through the activation function before the layer generates the output feature maps. The rectified linear unit (ReLU) activation function is a common activation function that can be defined as a piecewise linear function that will output the input signal if it is positive; otherwise, it will generate a zero value.
- Pooling layer
  To reduce the dimensions of the image, the pooling layer is responsible for combining the neighboring pixels into a single pixel and retrieving the optimal features of the input tensor. Down-sampling might be performed after the activation layer.

#### 3.4.1. CNN Model with Random Weights

To generate feature maps that can be classified with the employed classifier models in the next step, we first employed a CNN model with random weights and set convolutional layers as untrainable (Figure 3). In the proposed architecture, the first and second convolutional layers have 32 filters with dimensions of 3 × 3, and the ReLU is employed as the activation function. Therefore at the end, 32 features are extracted for each individual pixel for the input frame.

As we described before, a typical CNN architecture is composed of convolutional layers, activation layers, pooling layers, and a fully connected layer, which is responsible for classifying the generated features. The feature maps generated by the convolutional layers record the precise position of features. Generally, pooling is employed later to reduce the size of the generated feature map. Doing so can significantly reduce the number of parameters due to the dimension reduction procedure, which also helps to reduce training times.

However, in applications like semantic segmentation of anomalies, due to the selection of dominant features, the pooling layer cannot be employed. Reducing the dimensions of the input feature map comes with a disadvantage, as some components of an image can be shuffled through the pooling layer. In our proposed work, to perform the semantic segmentation task, we chose to collect features from each pixel of the input image; therefore, we did not employ the pooling layer.

#### 3.4.2. VGG-16 Model with Transfer Learning

The transfer learning approach in CNN-based feature extraction procedures has shown the potential for taking advantage of the weights of a pre-trained CNN network to perform feature extraction tasks in a new image dataset without the need to retrain the network to acquire new weights. This study was, thus, aimed at evaluating a transfer learning approach using a VGG16 architecture for semantic segmentation of thermal anomalies to identify overheated idlers in BC systems. The VGG16 architecture was first introduced in 2014 [56].

**Figure 4:**
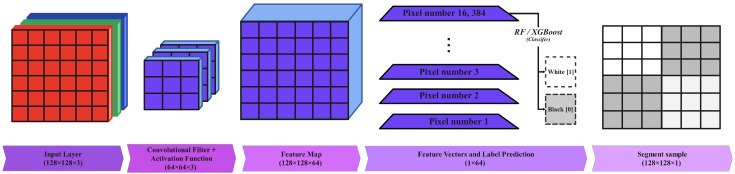
The proposed feature extraction and classification procedure using the VGG16 architecture.

In this work, we selected the VGG16 architecture as the backbone. We used the weight parameters from the VGG16 model, which was previously pre-trained on the ImageNet dataset (Figure 3). ImageNet was developed as a research project to provide a large database of images with annotations. The VGG16 model has already been trained on ImageNet, which comprises disparate categories of images. High-performance GPUs with millions of images and thousands of image categories were used to train the model [46]. In this work, we chose to only select the first and second convolutional layers of the VGG16 architecture to extract the desirable features, as we did not want to reduce the size of the feature maps by employing the pooling layer. Therefore, at the end, 64 features were extracted for each individual pixel for the input frame (Figure 4).

### 3.5. CNN Fusion with RF and XGBoost

The most efficient way to perform semantic segmentation tasks is to use the transfer learning approach. As we mentioned earlier, to improve the understanding of the employed classifier methods, we only chose to select the top level of the employed VGG16 model to generate the feature maps. Generally, at the latest stage of a CNN architecture, a fully connected layer is responsible for performing the feature classification task. In our work, we switched out the fully connected layers for XGBoost and RF models. The proposed approach based on CNN fusion with RF and XGBoost was studied to see how the extracted features in the convolutional layer can be used to classify input image pixels into the following two groups: background (cold area) and foreground (overheated idlers). To do so, the classifier models were separately trained using the extracted features from the training dataset. Afterward, the validation and test datasets were used to measure the final performance of the proposed approach in performing semantic segmentation tasks.

#### Classification Methods

In this paper, we studied the performance of two popular classifiers, namely RF and XGBoost, for the classification of the extracted features using the proposed convolutional filters.

RF is considered an ensemble approach that includes multiple decision trees to make predictions based on input features. Based on the feature maps generated by the CNNs, the decision trees of the RF determined the pixel label. To do so, the extracted features from CNN models were used to train the RF. Moreover, to improve the accuracy of the final decision, a majority vote between the decision trees is employed. Using multiple decision trees can reduce the risk of overfitting; therefore, in this work, each tree randomly selected a subset of features from the whole available data to improve the final prediction [57,58].

The XGBoost classifier works based on randomization techniques such as random subsamplingand column subsampling, which can speed up the training process and reduce the chance of the model overfitting. To reduce the computational cost, XGBoost ideally splits data into a compressed, pre-sorted, column-based system. This technique enables the classifier to simultaneously search for the optimal division of each considered attribute. Moreover, by employing the combined statistics, it can use a data percentile-based strategy to examine a limited subset of candidate splits and evaluate their gains. Here, as in the RF model, we trained the XGBoost using the features generated by the CNNs using the training dataset [43].

### 3.6. A PCA-K-Means Approach for Clustering the Segmented Thermal Anomalies

Thermal anomaly classification is a challenging task from the CM point of view, as segmented pixels in overheated areas might share similar patterns, like thermal anomaly distribution around the idler surface. The pixel-level segmentation method cannot provide information to characterize the thermal faults based on their degree of severity. In practice, to have a better understanding of the segmented samples, we need to deal with the high dimensionality of the processed samples, which not only directly affects the speed of the post-processing tasks but also makes it difficult for supervisors to visualize and understand the output results.

In this paper, we provide a dimension reduction and classification framework for classifying segmented masks generated in the previous section into different clusters based on the severity of faults in monitored idlers. To do so, we introduce a fully integrated classification and severity level assignment system for thermal fault characterization using a PCA-K-means approach.

#### 3.6.1. Centroid Calculation of Binary Masks

**Figure 5:**
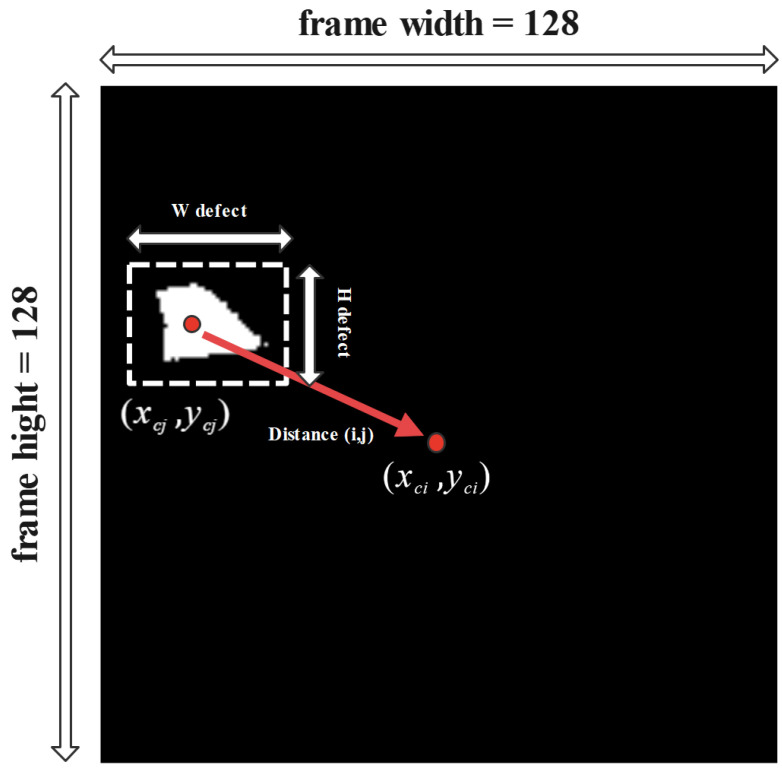
Illustration of centroid parameters.

The dissimilarity between the centers of identified thermal anomalies in the generated binary masks can affect the differentiation between pixels through the dimension reduction process. In other words, reducing the data dispersion in the binarized masks can lead to results that can be more accurately clustered into different classes by using the introduced PCA-K-means method. For this matter, first, the centroid location in generated binary masks is defined to relocate the masks into the frame center (Figure 5). For this matter, the following steps are followed:

According to Figure 5, *O*th represents the centroid of segmented thermal anomalies in each frame, and $y_{c}$ and $x_{c}$ represent the centers of the segmented areas. Moreover, $W_{object}$ and $H_{object}$ represent the width and height of the segmented areas, respectively [59].

(4) $$\left\{\begin{array}{c} y_{c}=y_{0}+\frac{H_{\mathrm{object}\;}}{2}\\ x_{c}=x_{0}+\frac{W_{\mathrm{object}\;}}{2} \end{array}\right.$$

(5) $$\left\{\begin{array}{c} W_{\mathrm{object}\;}=\max\arg\;\left(\mathrm{sum}\;\mathrm{pixels}\;=1\;\mathrm{in}\;\mathrm{each}\;\mathrm{row}\right)\\ H_{\mathrm{object}\;}=\max\arg\;\left(\mathrm{sum}\;\mathrm{pixels}\;=1\;\mathrm{in}\;\mathrm{each}\;\mathrm{column}\right) \end{array}\right.$$

(6) $$\left\{\begin{array}{c} y_{0}=\;\mathrm{location}\;\mathrm{of}\;\mathrm{the}\;\mathrm{starting}\;\mathrm{point}\;\mathrm{in}\;H_{\mathrm{object}\;}\\ x_{0}=\;\mathrm{location}\;\mathrm{of}\;\mathrm{the}\;\mathrm{starting}\;\mathrm{point}\;\mathrm{in}\;W_{\mathrm{object}\;} \end{array}\right.$$

The distance-wise dependencies in each frame can be calculated as follows:

(7) $$\;\mathrm{Distance}\;\left(i,j\right)=\frac{\sqrt[{2}]{{\left(x_{ci}-x_{cj}\right)}^{2}+{\left(y_{ci}-y_{cj}\right)}^{2}}}{\;\mathrm{Number}\;\mathrm{of}\;\mathrm{rows}\;\mathrm{of}\;\mathrm{the}\;\mathrm{image}\;}$$

**Figure 6:**
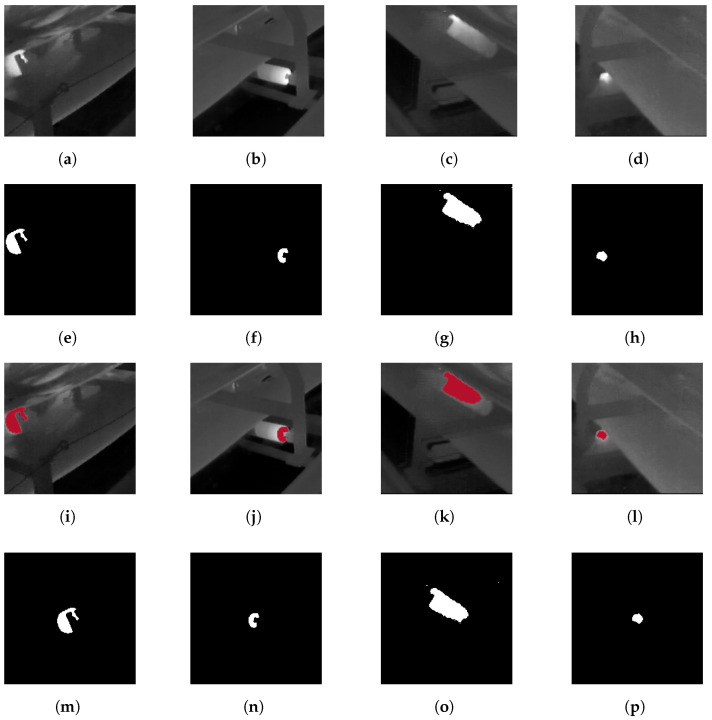
Examples of centralized segmented thermal defects based on the employed procedure. (**a**) Original ROI Sample 1, (**b**) Original ROI Sample 2, (**c**) Original ROI Sample 3, (**d**) Original ROI Sample 4, (**e**) Segmented label Sample 1, (**f**) Segmented label Sample 2, (**g**) Segmented label Sample 3, (**h**) Segmented label Sample 4, (**i**) Fault location Sample 1, (**j**) Fault location Sample 2, (**k**) Fault location Sample 3, (**l**) Fault location Sample 4, (**m**) Centralized label Sample 1, (**n**) Centralized label Sample 2, (**o**) Centralized label Sample 3, (**p**) Centralized label Sample 4.

Examples of the discussed procedure for centralizing the extracted defect areas for four different samples are shown in Figure 6.

**Figure 7:**
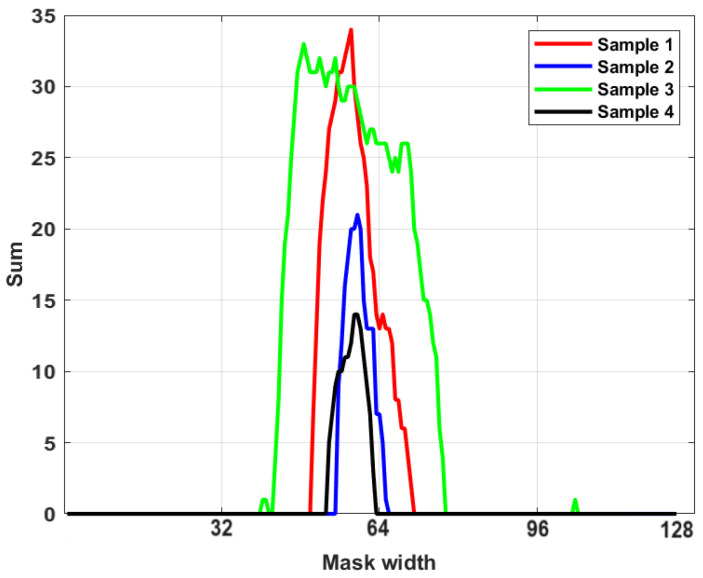
Profiles created from the row-column-wise pixel intensity summations with peaks corresponding to segmented defects.

Different feature extraction methodologies can be used to extract proper features from a segmented frame for dimension reduction and classification purposes. The shapes of the segmented areas in processed frames can be different due to the severity of the extracted thermal faults. In the binarized segmented map, the intensity value of the pixels varies from black (0) to white (1). To extract features, we scan the pixels’ columns to look for trends that can help pick out novelty. Therefore, the column-wise summation of each frame is generated and considered a feature for the performance of clustering tasks (Figure 7).

#### 3.6.2. PCA Method

Dimension reduction methods can be used to remove redundant and irrelevant features by reducing the data dimensionality [60]. The PCA method highlights the patterns in the data and improves the clustering results. It is an unsupervised dimension reduction method that does not require labels. This method generates sets of variables called principal components, where all the variables are orthogonal and each component is a linear combination of the reference components. Therefore, several top-ranking principal components can be selected to form a new feature space for the clustering of the samples.

PCA works by decomposing a covariance matrix into its eigenvectors. Covariance measures the degree of relationship between different variables in multivariate data. The mean value for the gathered data is subtracted in the first phase of the PCA method. The covariance matrix is then created, and the eigenvalues and eigenvectors of the covariance matrix are computed. Furthermore, the cumulative energy content of each eigenvector is calculated. In the following phase, to generate an ordered orthogonal basis, the eigenvectors are sorted based on the largest one, allowing us to express the data in terms of only a few orthogonal basis vectors rather than all the covariance matrix eigenvectors.

The PCA method consists of five main steps [61]. In the first step, the original data matrix can be listed as follows:

(8) $$X={\left(x_{ij}\right)}_{np}=\left[\begin{array}{ccc} x_{11} & \cdots & x_{1p}\\ \vdots & \vdots & \vdots \\ x_{n1} & \cdots & x_{np} \end{array}\right]$$

In our case, the $x_{ij}$ in the matrix (X) refers to profiles created from the row-column-wise pixel intensity summation with peaks corresponding to segmented defects calculated in the previous section. In this direction, *p* represents the selected column in the image (*n*), and $X_{np}$ defines the sum of the rows in column *p*.

In the second step, to eliminate the impact of dimensions, the Z-score standardization formula is used to standardize the original data as defined below [62].

(9) $$x_{ij}^{*}=\left(x_{ij}-\bar{x_{j}}\right)/s_{j}$$

In Equation (9), $x_{ij}^{*}$ is defined as the standard variable, and $\bar{x_{j}}$ is the average value for the $j_{th}$ indicator. Moreover, $s_{j}$ can be defined as the standard deviation for the $j_{th}$ indicator.

Through step three, the standardized correlation coefficient matrix (R) defines the correlation between the indicators in Equation (10) [63].

(10) $$R={\left(r_{ij}\right)}_{p*p}=\frac{1}{n-1}\sum_{t=1}^{n}x_{ti}^{*}\times x_{tj}^{*}\;\left(i,j=1,2,\cdots ,p\right)$$

In step four, the eigenvalues and eigenvectors of the correlation coefficient matrix (R) are defined to understand the number of principal components. $\lambda_{i}\left(i=1,2\cdots n\right)$ defines the eigenvalues of the correlation coefficient matrix (R), where the eigenvectors can be defined as $u_{i}\left(u_{i}=u_{i1},u_{i2},\cdots u_{in}\right)\left(i=1,2\cdots n\right)$. The principal component is represented as follows:

(11) $$F_{i}=u_{i1}x_{1}^{*}+u_{i2}x_{2}^{*}+\cdots +u_{in}x_{n}^{*}\;\left(i=1,2,\cdots ,n\right)$$

In Equation (11), $x_{i}^{*}$ is represented as the standardized indicator variable as $x_{i}^{*}=\left(x_{i}-\bar{x_{i}}\right)/s_{i}$.

In step five, to obtain an evaluation function, the obtained principal components are weighted and summed as shown below.

(12) $$F=\frac{\lambda_{1}}{\lambda_{1}+\lambda_{2}+\ldots +\lambda_{n}}F_{1}+\frac{\lambda_{2}}{\lambda_{1}+\lambda_{2}+\ldots +\lambda_{n}}F_{2}+\ldots +\frac{\lambda_{n}}{\lambda_{1}+\lambda_{2}+\ldots +\lambda_{n}}F_{n}$$

#### 3.6.3. K-Means Method

The PCA method cannot select a subset of features for distinguishing the classes. The extracted PCA components have no class label; therefore, an unsupervised clustering method is used for the feature classification problem. K-means is one of the most popular unsupervised learning techniques for dealing with the clustering problem. The approach follows a simple procedure for classifying a given dataset using a defined number of clusters (assuming k clusters) [64].

The K-means method is divided into two parts. In the first step, it computes the k centroid, and in the second phase, it assigns each data point to the cluster with the closest centroid to it. In our case study, the data point was defined as the first three components extracted using the PCA method introduced in the previous section. In other words, instead of analyzing the 128 data points defined by profiles created from the row-column-wise pixel intensity summation with peaks corresponding to segmented defects, we reduce the number of data points to three. By performing this procedure, we could easily visualize thermal defects using three coordinates and apply K-means to the data points to efficiently cluster them into different groups. The Euclidean distance is one of the most commonly used methods for determining the distance to the nearest centroid. Once the grouping is complete, it recalculates the new centroid of each cluster, and based on that selected centroid, it calculates a new Euclidean distance between each center and each data point and assigns the cluster points with the shortest Euclidean distance. Each partition’s cluster is defined by its member objects and centroid. The centroid of any cluster is the place at which the total distance from all objects in that cluster is the smallest. Therefore, K-means is an iterative technique that minimizes the sum of distances between each item and its cluster centroid over all clusters. In our study, to reveal the underlying patterns in segmented thermal faults, the most informative PCA components were chosen for clustering by the K-means method.

## 4. Evaluation Metrics

Assessing the efficacy of semantic segmentation methods can be challenging, as it necessitates evaluating both pixel-level classification and localization. The Jaccard index and mean Jacard index are standard metrics that are highly efficient in evaluating semantic segmentation models [65,66].

To explain the metrics, first, we assume a total of k+1 classes, including a void class or background from $L_{0}$ to $L_{K}$. Moreover, we define $p_{ij}$ as the number of pixels in class *i* inferred to belong to class *j*. In this direction, we can define $p_{ii}$ as the number of true positives, while $p_{ij}$ and $p_{ji}$ are interpreted as false positives and false negatives, respectively [66,67].

**Figure 8:**
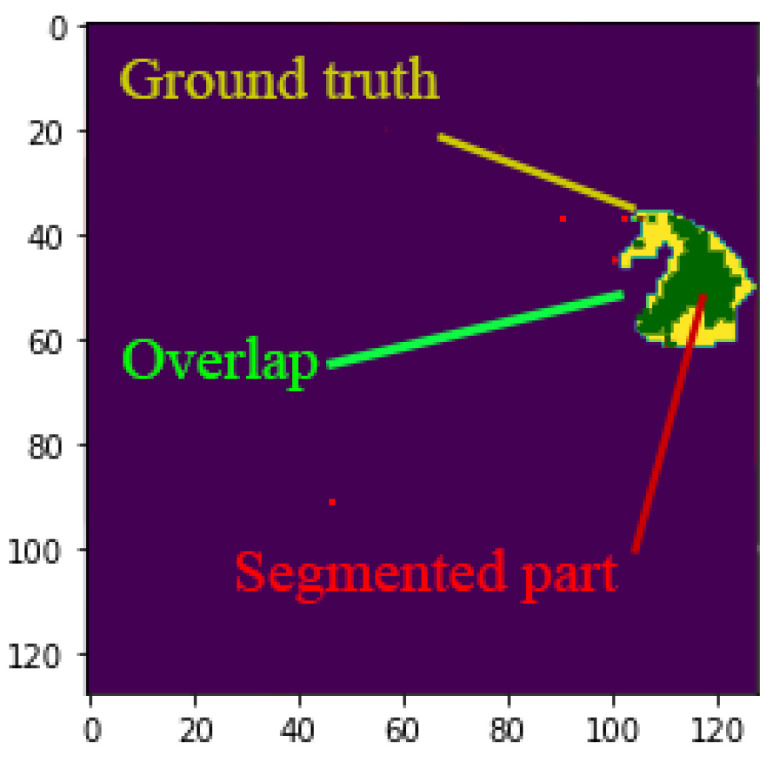
Given two sets, namely the ground truth (yellow section) and segmented parts (red parts), the Jaccard index is defined as the size of the intersection of yellow and red section (indicated by green).

The Jaccard index works based on the calculation of the intersection and union of two sets—in our case, the ground truth and our predicted label (Figure 8). The ration can be defined as the number of true positives (TP) or, in other words, the intersection over the sum of TP, false positives (FP), and false negatives (FN), as follows:

(13) $$\mathrm{Jaccard}\;\mathrm{index}=\frac{TP}{TP+FP+FN}$$

The mean Jaccard index can be defined as the per-class basis, then averaged, as defined below.

(14) $$\mathrm{Mean}\;\mathrm{Jaccard}\;\mathrm{index}=\frac{1}{k+1}\sum_{i=0}^{k}\frac{p_{ii}}{\sum_{j=0}^{k}p_{ij}+\sum_{j=0}^{k}p_{ji}-p_{ii}}$$

In Section 5.2, to evaluate the performance of our proposed dimension reduction and classification approach, we employed accuracy as the performance metric. The quantity of true negatives (TN) can have a significant impact on this metric, which can give us a good idea of the proportion of accurately clustered samples, as shown in Equation (15) [35].

(15) $$\;\mathrm{Accuracy}\;=\frac{\left(TP+TN\right)}{\left(TP+FN)+(FP+TN\right)}\;$$

## 5. Results and Discussion

The whole series of experiments and data collection experiments are carefully described in our previous work [17,35,41]. The IR videos were captured by a FLIR T640 camera (FLIR Systems, Wilsonville, OR, USA), which has a resolution of 640 × 480 pixels, a maximum frame rate of 30 frames per second, and a thermal sensitivity of 0.035 °C. The FLIR thermal studio suite was used to view and post-process the thermographic results. To process the acquired information from different sensors, we employed an laptop featuring an eight-core Ryzen 7 5800H (AMD Inc., Santa Clara, CA, USA) processor and an RTX 3060 graphics card with 6 GB of GDDR5 VRAM (Nvidia Corporation, Santa Clara, CA, USA) and 16 GB of DDR4 RAM (Kingston Technology, Fountain Valley, CA, USA).

We used two different datasets to train, validate, and test the studied semantic segmentation models. In this direction, dataset one includes 179 positive augmented samples that were captured from the BC while the mobile robot moved forward (FW) alongside the conveyor system. This dataset is split into 80% for training and 20% for validation of the models. On the other hand, in dataset number two, the mobile robot captured the IR image samples from the same conveying system while moving backward (BW), including 163 positive samples that were not augmented. This dataset was used to test the model’s performance using unseen samples.

### 5.1. Semantic Segmentation Models

The Jaccard index is an important performance metric that directly measures the degree of deference between the predicted segmented masks and the actual boundary. Through experiments, we first measured the performance of the employed CNN architecture with random weights in the correct segmentation of the overheated area using RF and XGBoost as pixel classification models. Moreover, we studied the performance of the pre-trained VGG16 architecture as the feature extraction solution, together with RF and XGBoost, in the same direction.

We set the experimental parameters of XGBoost and RF carefully to balance the resources used while achieving good performance, guided by the time complexity of our models using training and validation datasets. The values and meanings of the selected hyperparameters for RF and XGBoost methods are presented in Table 2.

To compare this study with our previous work, different variations of the U-Net architecture, including base U-Net [18], attention U-Net [68], and attention residual (ARes) U-Net [69], were employed to be compared with the proposed CNN models in the performance of semantic segmentation tasks [17]. In this study, binary cross-entropy (BCE) was employed as a loss function to train the U-Net models [70]. Table 3 and Table 4 compare the performance metrics of the studied methods.

In this paper, we first analyze the performance of non-transfer learning approaches for the feature extraction process using the proposed CNN architecture. The results show that the mean Jaccard index scores for CNN models with random weights using RF and XGBoost were 0.7974 and 0.7953, respectively, on the test dataset. In this direction, we studied the pre-trained VGG16 performance metrics as well, where the mean Jaccard index score of the employed VGG16 with RF and XGBoost was above 0.9050 using the employed classification approaches on the test dataset. One can notice that the use of XGBoost can sufficiently reduce the training time compared to the RF model while generating masks with the same level of accuracy.

**Figure 9:**
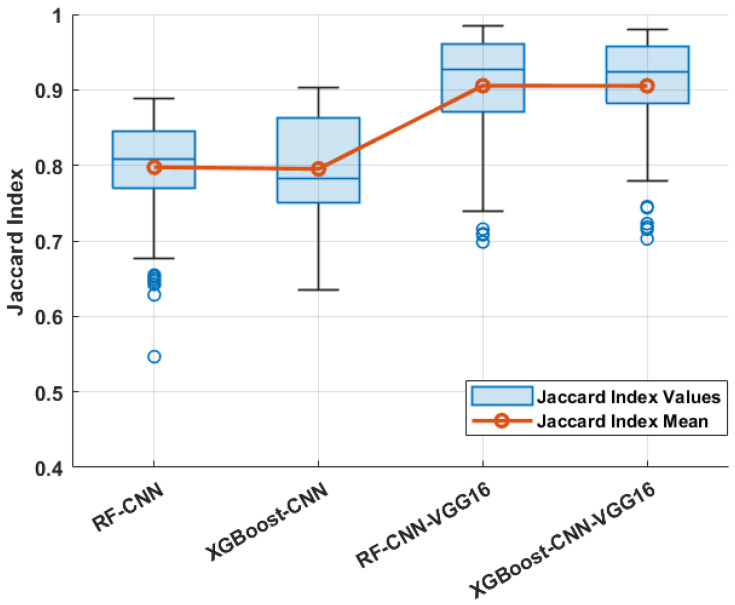
Box plot of studied CNN models on test samples. The middle line of the box shows the median in the figure; the boxes are extended to the lower and upper quartiles, the whiskers are the most extreme values, and the blue dots denote the outliers.

**Figure 10:**
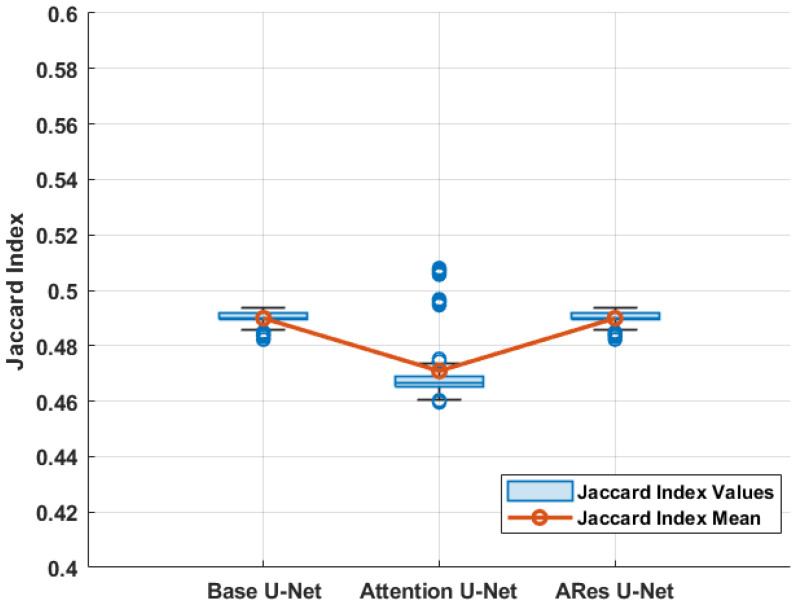
Box plot of studied U-Net models on test samples.

After analyzing the above results, it can be found that the employed VGG-16 model with pre-trained weights could significantly improve the segmentation results while reducing the training time. Our results indicate that the VGG-16 model already trained on the ImageNet dataset, together with the XGBoost classifier, can provide the best performance when it comes to datasets with a limited number of training samples and complex scenes. Figure 9 and Figure 10 show box plots for each of the seven studied models using the Jacard index score as the performance metric.

**Figure 11:**
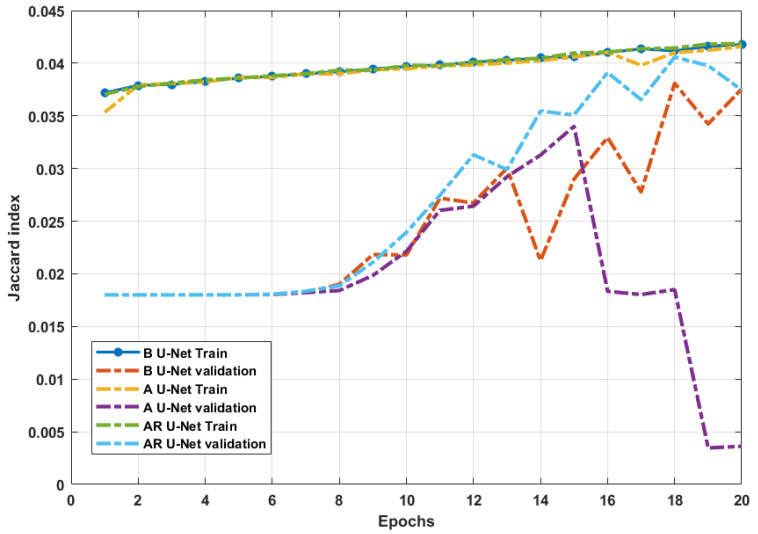
Evaluating the performances of the U-Net models through training and evaluation in each epoch.

As a deeper network than the employed VGG-16 architecture, the studied U-Net models need to achieve better performance in theory, but the experimental results are just the opposite. The main reason for this issue is the number of trainable parameters as a result of the complexity of U-Net-based CNN architectures. Considering the nature of our dataset, we can notice that due to the limited number of samples for training, the U-Net architecture did not have access to enough information through the training epochs (Figure 11). It is worth mentioning that the prediction speed of all the studied models was below 0.5s after the initial training.

**Figure 12:**
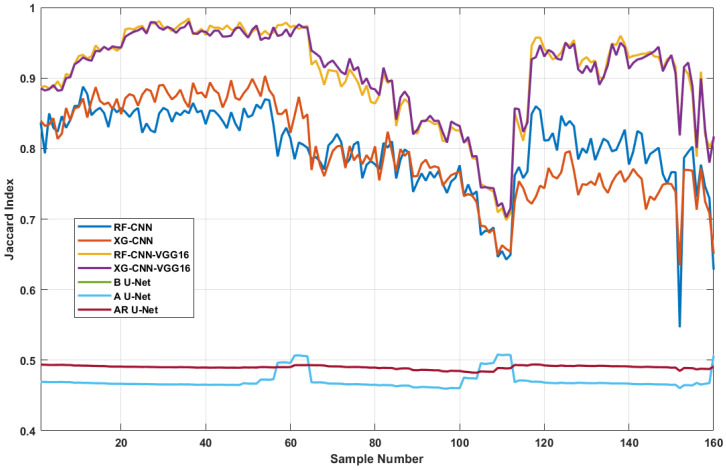
Analyzing the Jaccard index score over each test sample in the test dataset to compare the model trends in performing semantic segmentation tasks.

Although the methods proposed in this paper achieved promising results for the identification of overheated idlers, they achieved poor performance in some specific cases. We attempted to analyze the reasons for the unsatisfactory segmentation results; therefore, we studied the calculated Jaccard index value of each sample in the test dataset (Figure 12).

One can notice that there is a sudden drop in Jaccard index scores between sample numbers 100 and 120. Our investigation shows that the mentioned samples were blurry and not sharp enough to be correctly segmented by the employed model. During the experiments, the mobile robot followed a straight line alongside the BC systems, continuously capturing IR videos from the idlers. The IR camera was installed on a robotic hand 1.5 m above the inspection robot, which provided the camera with a good point of view over the BC system. Throughout the conducted experiments, the mobile robot maintained a constant speed to minimize the risk of sudden vibrations affecting the equipped camera on the inspection robot. However, considering the uneven ground conditions at mining sites, the equipped camera can experience sudden shakes, which might cause it to capture blurry images.

Our further analysis showed that sample numbers 20 to 40 corresponded to images captured under good conditions that were relatively sharp; however, sample 100 to 120 were blurry. On average, the mean Jaccard index in ideal samples was above 0.9; however, this score dropped to below 0.75 in blurry ones according to Figure 12.

We consider that several reasons are led to poor segmentation. (1) Some false defects are similar to defects, resulting in false detection. (2) Some defects have the same texture as the surrounding environment, which makes them difficult to identify. (3) The shape of some defects is so complex that only a small part can be segmented.

**Figure 13:**
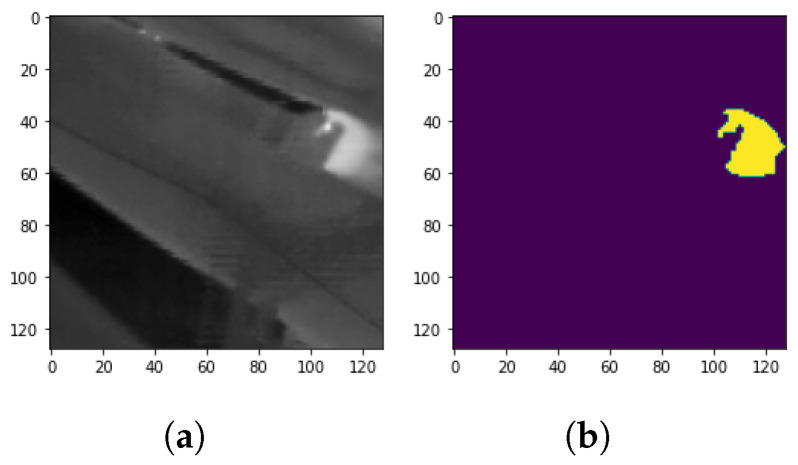
A selected complex sample to demonstrate the performance of the studied methods. (**a**) Sample one. (**b**) Ground-truth label Sample one.

**Figure 14:**
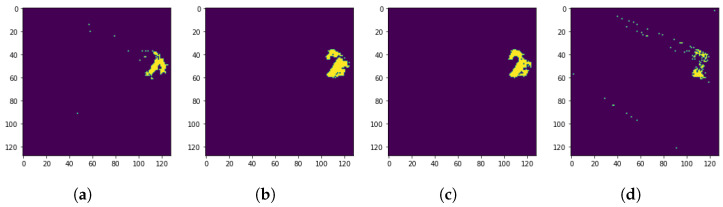
Comparison of studied model results in correct segmentation of an overheated idler in a complex sample. (**a**) RF-CNN Jacard index: 0.6878. (**b**) XGBoost-CNN Jacard index: 0.8008. (**c**) RF-CNN-VGG16 Jacard index: 0.7884. (**d**) XGBoost-CNN-VGG16 Jacard index: 0.6248.

For an intuitive explanation, Figure 13 displays an example of where the employed models generate labels with low Jaccard index scores. In this example, due to camera shake, one can notice that the captured overheated idler seems blurry. As long as the studied models were trained on cases where faulty idlers were captured sharply, we can notice that all models had problems correctly segmenting the pixels that represent the overheated areas (Figure 14).

While segmentation accuracy (Jaccard index score) is a critical metric, it is not the only factor that should be considered when comparing the semantic segmentation methods. Indeed, training time, evaluation speed, and model complexity are some other vital factors to consider from the perspective of real-world training and model deployment, both in research and industrial practices. Small performance improvements may not be worthwhile in certain applications if they are obtained at the cost of significantly increased complexity and reduced speed. In this case, while the pre-trained VGG16 architecture with XGBoost as a classifier achieves a lower score in comparison to the same architecture with RF as a classifier, it is still comparable to the majority of the other architectures across all datasets and should be considered the best approach.

### 5.2. Fault Clustering Model

The segmented frames that were generated in the previous step do not have any labels; therefore, it could be difficult for the supervisor to distinguish and study the faulty idlers from the degree-of-severity point of view. Clustering the overheated idlers might provide us with information to rapidly recognize the most important ones that need to be immediately replaced and also let us find faults that share similar patterns. Identifying a group of faults that indicate a similar pattern can help the supervisors investigate the main reason behind idler bearing degradation over time.

Considering the high performance of the employed method in semantic segmentation tasks, the frames where the model identifies the hotspots are considered faulty frames. To accomplish this, we first use the method described in Section 3.6.1 to centralize the mask after saving the labels generated for dataset number two by the RF-CNN-VGG16 model, as it achieved the best performance among the studied methods. The frames with signs of fault were related to four different idlers. As the frames were captured continuously, the faults related to each idler were distinguishable in the sequence of the processed frames. Afterward, we calculated the column-wise summation of each frame as a feature. To perform the dimension reduction procedure and reduce the size of the extracted feature, we calculated the percentage of variance explained by each of the first 10 principal components.

**Figure 15:**
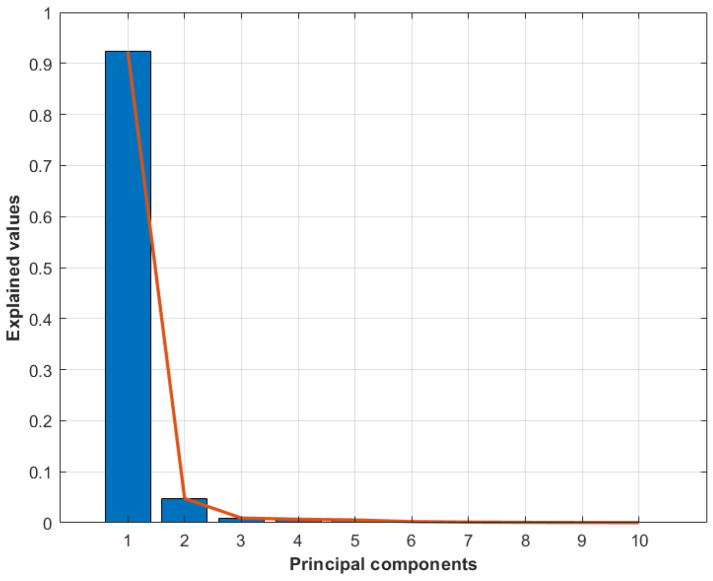
Scree graph showing the percentage of variance explained by each of the first 10 principal components.

A scree plot was used to check the optimal number of principal components required to explain a sizable amount of variation in the data. From Figure 15, it can be seen that almost 99% of the variability is explained by three principal components, and the remaining principal component explains less than 1% of the variability. Therefore, three principal components were chosen for further analysis and classification.

**Figure 16:**
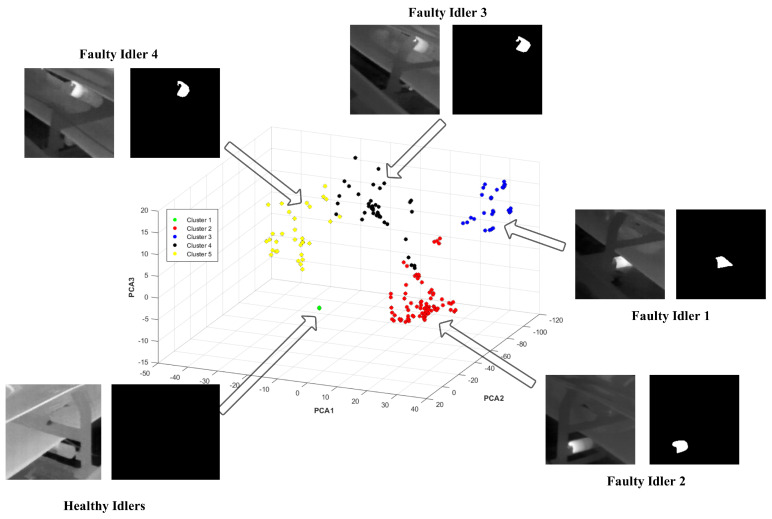
PCA plot showing the first three principal components of the dataset.

The performance of the proposed PCA-K-means method in dimension reduction and clustering procedures was examined on the segmented samples from dataset 2 (the test dataset) from the previous section. In this direction, 163 samples containing thermal anomalies, together with 20 healthy samples, were subjected to dimension reduction and clustering procedures and are finally presented as Figure 16.

The accuracy of the clustering procedure for each cluster is presented in Table 5. The main reason to perform dimension reduction and cluster the faults in Figure 16 is to first reduce the size of the gathered data. In the industrial big data processing approach, where hundreds of idlers are regularly inspected, it can be beneficial for supervisors to reduce the size of the gathered data and visualize them in a simple form, as we have presented in this paper.

In this direction, we investigated the effectiveness of the studied clustering method on different thermal defects, as presented. In Table 5, we try to demonstrate how idler faults can share a similar pattern. The experimental results demonstrated that the combination of PCA and the K-means clustering methods is an efficient way of clustering thermal anomalies in highly overlapped IR image data.

The accuracy here indicates how the K-means method could distinguish and cluster the faults. However, as long as some faults can share the same features in terms of shape and size, it is normal that they are clustered in other fault classes. The accuracy scores of the classification of faulty idler 1, faulty idler 2, faulty idler 3, faulty idler 4, and healthy idlers are 77.65%, 87.87%, 70.37%, 100%, and 100%, respectively. As we can see here, at least 70% of faults were correctly clustered in the true class.

## 6. Conclusions

The early detection and precise localization of overheated idlers play a pivotal role in averting unforeseen shutdowns in BC systems. Manual CM methods, particularly in harsh environments, are susceptible to potential inaccuracies. Recognizing the likelihood of human errors in BC idler CM, the imperative for an automated robotics-based approach becomes evident. Given the challenges and costs associated with establishing an online network at mining sites, mobile inspection robots offer an advantage for an offline data gathering process. We downloaded the captured data from the experiment to a local computer for further processing. In doing so, we did not have to install a special network system at the studied mining site to capture the data, which considerably reduces the cost of employing such a system in real-life scenarios. Furthermore, the mobile inspection robot can begin analyzing the gathered data immediately after completing its inspection tasks. Analyzing the data in an offline manner reduces the need for equipment for the installation of mobile robots with complex computers and lets us employ a complex deep learning model using the computers in the control room.

In this study, we showcase the utility of the VGG16 architecture as a feature extractor, complemented by XGBoost and RF as feature classifiers, to effectively discern thermal anomalies in IR image samples. Our approach demonstrates its efficacy in semantic segmentation tasks, particularly within highly imbalanced datasets with a limited number of positive samples. From an accuracy perspective, experimental results confirm the superiority of the suggested VGG16 architecture with RF as a feature classifier, achieving a commendable 0.9054 score on the mean Jaccard index metric. On the other hand, considering the computational time perspective and accuracy together, the VGG16 architecture with XGBoost as a classifier demonstrates superiority over the other compared models with training times below 15 s.

In addition, we propose a dimension reduction and classification strategy based on PCA-K-means to cluster segmented frames generated by the employed segmentation models. Our proposed approach proves successful in reducing dimensions and accurately classifying unlabeled thermal defects based on severity in over 70% of the examined cases.

Recognizing the paramount importance of prompt and accurate identification and classification of thermal defects in BC idlers for the enhancement of BC operation safety, our future work includes the incorporation of fusion methods leveraging additional data sources such as acoustic signals and RGB images. These sources aim to assist in early detection of potential faults in BC idlers.

There are a few key points that we intend to improve in our future research. First, considering the existence of blurry samples in captured IR images, the idea of employing advanced pre-processing approaches such as artificial intelligence-based super-resolution or deburring methods can be beneficial to improve the overall quality of samples. Moreover, for efficient clustering data that can share similar patterns, it could be beneficial to study more advanced dimension reduction and clustering methods such as t-distributed stochastic neighbor embedding (t-SNE), uniform manifold approximation and projection (UMAP), and density-based spatial clustering of applications with noise (DBSCAN) compared to the method proposed in this paper. Furthermore, we intend to use a information fusion approach to capture LiDAR data using the mobile inspection robot. The gathering of point clouds can enable us to not only detect faulty idlers but also tag their geographical locations.

## Figures and Tables

**Table 1:** Comparison of the portions of positive and negative samples that were extracted frames from captured IR videos.

	Dataset 1	Dataset 2
Total number of extracted frames	6135	6135
Number of inspected idlers	130	130
Percentage of positive cases	5.21%	6.67%
Percentage of negative cases	94.78%	93.32%

**Table 2:** Hyperparameters of the employed RF and XGBoost methods and their values.

Model	Hyperparameters	Meaning	Values
RF	$n_{tree}$	Number of trees used in the forest	50
	$m_{try}$	Number of random variables used in each tree	42
XGBoost	Learning rate	Shrinkage coefficient of each tree	0.3
	Maximum tree depth	Maximum depth of a tree	6
	Subsample ratio	Subsample ratio of training samples	1
	Column subsample ratio	Subsample ratio of columns for tree construction	1
	Maximum delta step	Maximum depth of a tree	0
	Gamma	Minimum loss reduction required to make a further partition	0

**Table 3:** The experimental results of the studied semantic segmentation methods.

Model	Epochs	Trainable Parameters	Training Time	Mean Jaccard Index	
				**Validation-BC 1 (FW)**	**Test Set-BC 1 (BW)**
RF-CNN	-	-	429 s	0.8025	0.7974
XGBoost-CNN	-	-	**10 s**	0.8026	0.7953
RF-CNN-VGG16	-	-	270 s	**0.9439**	**0.9054**
XGBoost-CNN-VGG16	-	-	13 s	0.9364	0.9052
Base U-Net	20	31 M	80 s	0.4615	0.4899
Attention U-Net	20	37 M	102 s	0.4764	0.4708
ARes U-Net	20	39 M	118 s	0.5002	0.4899

The bold number indicate the highest score.

**Table 4:** Statistical analysis of studied models.

Model	Mean	Median	Minimum	Standard Deviation
RF-CNN	0.7975	0.8079	0.5466	0.0577
XGBoost-CNN	0.7954	0.7833	0.6343	0.0667
RF-CNN-VGG16	0.9054	0.9263	0.6985	0.0668
XGBoost-CNN-VGG16	0.9053	0.9232	0.7026	0.0639
Base U-Net	0.4899	0.4901	0.4822	0.0026
Attention U-Net	0.4709	0.4667	0.4596	0.0123
ARes U-Net	0.4899	0.4901	0.4822	0.0026

**Table 5:** Performance of proposed PCA-K-means approach in true clustering of segmented samples.

Studied Case	Cluster 1	Cluster 2	Cluster 3	Cluster 4	Cluster 5	Accuracy
Faulty Idler 1	0%	21.27%	77.65%	0%	0%	77.65%
Faulty Idler 2	0%	87.87%	0%	14.12%	0%	87.87%
Faulty Idler 3	0%	29.63%	0%	70.37%	0%	70.37%
Faulty Idler 4	0%	0%	0%	0%	100%	100%
Healthy Idlers	100%	0%	0%	0%	0%	100%

## Data Availability

Archived datasets cannot be accessed publicly according to the NDA agreement signed by the authors.
